# A Novel Approach to Solve the “Missing Marker Problem” in Marker-Based Motion Analysis That Exploits the Segment Coordination Patterns in Multi-Limb Motion Data

**DOI:** 10.1371/journal.pone.0078689

**Published:** 2013-10-30

**Authors:** Peter Andreas Federolf

**Affiliations:** Department of Physical Performance, Norwegian School of Sport Sciences, Oslo, Norway; University of California, Merced, United States of America

## Abstract

Marker-based human motion analysis is an important tool in clinical research and in many practical applications. Missing marker information caused by occlusions or a marker falling off is a common problem impairing data quality. The current paper proposes a conceptually new gap filling algorithm and presents results from a proof-of-principle analysis. The underlying idea of the proposed algorithm was that a multitude of internal and external constraints govern human motion and lead to a highly subject-specific movement pattern in which all motion variables are intercorrelated in a specific way. Two principal component analyses were used to determine how the coordinates of a marker with gaps correlated with the coordinates of the other, gap-free markers. Missing marker data could then be reconstructed through a series of coordinate transformations. The proposed algorithm was tested by reconstructing artificially created gaps in a 20-step walking trial and in an 18-s one-leg balance trial. The measurement accuracy’s dependence on the marker position, the length of the gap, and other parameters were evaluated. Even if only 2 steps of walking or 1.8 s of postural sway (10% of the whole marker data) were provided as input in the current study, the reconstructed marker trajectory differed on average no more than 11 mm from the originally measured trajectory. The reconstructed result improved further, on average, to distances below 5 mm if the marker trajectory was available more than 50% of the trial. The results of this proof-of-principle analysis supported the assumption that missing marker information can be reconstructed from the intercorrelations between marker coordinates, provided that sufficient data with complete marker information is available. Estimating missing information cannot be avoided entirely in many situations in human motion analysis. For some of these situations, the proposed reconstruction method may provide a better solution than what is currently available.

## Introduction

Marker-based analysis of movement has become the basis for a broad spectrum of research and practical application areas ranging from clinical gait analysis, sports biomechanics, military sciences, computer vision, to video game development and other applications. Loss of marker information due to a marker falling off or due to marker occlusion is a frequent challenge in marker-based motion capture. The resultant data gaps compromise the accuracy of the analysis [1] or may pose a practical challenge, for instance, when post-processing includes fitting the marker configuration to a biomechanical model that requires the missing marker.

Standard motion tracking software, such as EVaRT (Motion Analysis Corporation, Santa Rosa CA, USA), Qualisys Track Manager (Qualisys AB, Gothenburg, Sweden), or Vicon (Oxford Metrics, Limited, Oxford, England), usually include basic gap filling methods based on spline interpolation or on reconstruction of markers in a local segment coordinate system. In many situations these tools provide a satisfactory solution to the missing marker problem. However, interpolation methods tend to be inaccurate if the gap is too long (e.g. 200 ms [2]) or when the reconstructed trajectory goes through a local extremum. Furthermore, they are not applicable if the gap is at the beginning or end of the measurement. Reconstruction from a local segment coordinate system assumes that distances between markers on this segment are conserved and requires three additional markers on the segment.

Several additional methods have therefore been developed in recent years, for instance, based on modified Kalman filters [3]–[6], Taylor series [7] or based on data-driven pattern recognition methods [8]–[11]. Kalman-filter approaches are well suited for real-time applications, however, their accuracy deteriorates if a marker is missing for an extended period. Data-driven pattern recognition methods typically use a database of similar motions, identify similar poses (e.g. through a nearest-neighbour algorithm), and improve their prediction with additional optimization routines [8]. These methods often allow prediction of several missing markers, however, their limitations include the need for an additional dataset of similar movements.

This technical note proposes an alternative technique to reconstruct missing marker information and reports results of a proof-of-principle analysis evaluating its performance when reconstructing marker data recorded in walking or balancing trials.

## Methods

### Conceptual Outline

Human (or animal) motion is a complex, neural-system-controlled, multi-body movement. It is characterized by a large number of mechanical degrees of freedom creating an abundance of possible solutions for any given motion task [12]. However, a multitude of internal and external constraints govern and restrict every movement pattern [13], [14] leading to a dramatic reduction in the actual degrees of freedom and facilitation of a self-organizing control structure (“dynamic systems theory” [15], [16]). These constraints arise, among other causes and mechanisms, from mechanical principles and requirements (e.g. stability), from anatomical properties of the moving organism [17], from the individual motor learning history and experiences, from psychological phenomena (e.g. emotional states [18]), or from influencing environmental factors (e.g. ground surface properties, footwear, clothing, etc. [17], [19]–[21]). Consequently, despite the vast abundance of potential solutions for a given task, usually a distinct, highly individual and characteristic movement pattern emerges, in which the movements of the individual body segments are intercorrelated in a specific way.

Principal component analysis (PCA) and other multivariate analysis methods can be used to determine the interrelation between kinematic movement variables [22], [23], thus characterizing the specific solution space that emerges in a given set of constraints [24]. The gap filling algorithm suggested in the current paper is based on the underlying idea that missing variables may be reconstructed if: (i) the interrelations between observed variables describing a movement are known, for instance through a PCA, and: (ii) if sufficient information from other variables is available to determine what state of motion a subject is in.

PCA applied to posture vectors incorporating the spatial coordinates of all markers of a given marker set yields a set of principal component vectors (PC vectors) which form a basis in the vector space spanned by the posture vectors [22], [23], [25]–[28]. Transforming the data, originally expressed in a coordinate system spanned by the measured marker coordinates into the system spanned by the PC-vectors, allows the expression of the complex multi-segment movement of the subject as a combination of one-dimensional movement components [22], [23], [28], also called “*principal movements*” [25]–[27]. Each *principal movement* quantifies a specific pattern of change in the positions of all markers, where the movement of each individual marker is correlated with the movements of the other markers. In other words, each *principal movement* can be viewed as a specific, one-dimensional representation of the internal constraints that govern the interrelations between the marker movements. The combination of all principal movements quantifying a movement can then be viewed as a representation of all internal constraints that lead to the specific, characteristic movement pattern of an individual in a given situation.

A PCA calculated for a subset of the data where all markers are available thus provides the information of how the position of the missing marker correlates with the positions of the other markers – and thus, how it can be reconstructed if the other marker positions are known. The challenge is then, how incomplete data (due to a missing marker) can be transformed into this PCA space. This was solved by first calculating a second PCA for the same subset but with the coordinates of the missing marker zeroed and then a transformation matrix **T** between the two PC-vector basis systems.

### Implementation Steps

The observed data were arranged in a *n x 3m*-matrix, **M**, with each line representing one time frame (where *n* is the total number of observed time frames) and each column representing a marker coordinate (m the number of markers). Some entries in **M** are missing.

As a first step, the marker coordinates in **M** were centered by determining for each frame the mean position of all markers that are available throughout the whole dataset. This trajectory of mean marker positions was subtracted from all markers in each frame. After the reconstruction procedure, the mean trajectory was added back to retrieve the original, non-centered marker trajectories.

To determine the reconstruction transformation, two smaller matrices **N** were created from the matrix **M**. First, all line vectors (time frames) containing a full set of marker coordinates were used to form a new *n^no_gaps^ x 3m*-matrix, **N^no_gaps^** (*n^no_gaps^* < *n*). Then, a second *n^no_gaps^ x 3m*-matrix, **N^zeros^**, was created as a copy of **N^no_gaps^**, except that those marker coordinates where **M** had gaps, were replaced by zeros.

Two principal component analyses were conducted separately for **N^no_gaps^** and **N^zeros^**. The PC-vectors obtained for **N^no_gaps^** form a basis {**PC^no_gaps^**} for principal movements based on all marker coordinates. The PC-vectors obtained for **N^zeros^** form a basis {**PC^zeros^**} for principal movements that consider no information from those markers that had gaps. Both basis systems, {**PC^no_gaps^**} and {**PC^zeros^**}, are 3m x 3m-matrices. Hence, a transformation matrix **T** facilitating a transformation of the data from the {**PC^zeros^**}-system into the {**PC^no_gaps^**}-system could be determined.

A matrix **R** with reconstructed marker coordinates was obtained by: (1) creating a copy **M^zeros^** of **M** where columns from **M** containing gaps were replaced by zeros; (2) transforming the dataset **M^zeros^** into the {PC**^zeros^**}-coordinates; (3) multiplying the result by **T** to obtain the dataset in the {PC**^no_gaps^**}-system; and (4) transforming the data back into the original coordinate system defined by the complete set of marker coordinates:

R  =  M^zeros^ PC^zeros^ T (PC^no_gaps^)^−1^

Without a normalization of the **M** and **N** matrices, the {**PC**}-systems are dominated by marker coordinates with large motion amplitudes (typically markers on the extremities). Depending on the marker that needs to be reconstructed, this can be an advantage or a disadvantage. To better standardize the reconstruction algorithm, the mean marker positions calculated for each column of **N^no_gaps^** were subtracted from all vectors in the **M** and **N** matrices and the result was normalized to unit variance. Assuming that marker coordinates to be reconstructed correlate strongest with the coordinates of adjacent markers, the vectors in **M** and **N** were additionally multiplied with a weight vector **w**, in which the closest markers were assigned a weight of 10 and second nearest neighbors a weight of 5. For example, if a knee marker was reconstructed, a weight of 10 was used for markers on the thigh and shin segments and a weight of 5 for the ankle and trochanter/ASIS markers. The reconstruction result could be improved further by reducing the number of PC-vectors used in the reconstruction from 3*m* (dimension of the {**PC**}-systems) to a lower number. The underlying idea for this modification was that measurement noise affecting the marker trajectories is typically not correlated between different markers. The noise is therefore predominantly represented in higher order PC-components (small signal-to-noise ratio), while the (correlated) motion data is represented in lower order PC-components (high signal-to-noise ratio). In the motion data analyzed in the current study, the reconstruction was based on 40 PC-vectors, which appeared to be a reasonable compromise between using as much of the information as possible and reducing the influence of noise on the reconstruction. All processing steps were implemented in a Matlab-function (Matlab®, The MathWorks Inc., Natic, MA, USA). A copy of the Matlab-code of the reconstruction algorithm has been attached to this manuscript as supplementary material.

### Data Selected for the Proof-of-Principle Analysis

Two essential types of human movement, the cyclic motion of walking on a treadmill and the postural sway motion of balancing in a one-leg stance, were selected for the proof-of-principle analysis in this study. Each movement had been conducted by one healthy young volunteer as a trial for other studies [26], [29] that had been approved by the Conjoint Health Research Ethics Board at the University of Calgary. The volunteers provided informed written consent prior to participating.

The subjects’ movement patterns were recorded with a full-body marker set consisting of 37 markers. The markers were positioned on the volunteers in accordance with the Vicon Plug-In-Gait marker set (Oxford Metrics). Specifically, markers were placed on the left (*L…*) and right (*R…*) foot on the second metatarsal head (*LTOE, RTOE*); posterior on the calcaneous (*LHEE, RHEE*); on the lateral malleolus (*LANK, RANK*); shank (*LTIB, RTIB*); lateral epicondyle of the knee (*LKNE, RKNE*); thigh (*LTHI, RTHI*); anterior and posterior superior iliac spine (*LASI, RASI, LPSI, RPSI*); 7^th^ cervical and 10^th^ thoracic vertebrae (*C7, T10*); sternum (*STRN*); clavicle (*CLAV*); right scapula (*RBAK*), superior on the acromio-clavicular joint (*LSHO, RSHO*), upper arm (*LUPA, RUPA*); lateral epicondyle of the elbow joint (*LELB, RELB*); forearm (*LFRA, RFRA*); both sides of the wrist joint (*LWRA, RWRA, LWRB, RWRB*); and four markers were attached to a head band worn by the volunteer (*LFHD, RFHD, LBHD, RBHD*).

All markers were spherical, retro-reflective markers with a cross section of 20 mm. The 3D-positions of these markers were determined with a frame rate of 240 Hz using a standard eight-camera motion capture system (Motion Analysis Corporation, Santa Rosa, CA, USA). The average 3D-residual of the camera calibration was 0.5 mm. The coordinate system was oriented such that the X-coordinate pointed in the anterior direction, the Y-coordinate in the medio-lateral direction, and the Z-coordinate in the vertical direction with respect to the volunteer. Post-processing (marker identification) was conducted using Eva Real-Time Software (EVaRT, Motion Analysis Corporation, Santa Rosa, CA, USA). The selected data sequences had no gaps and no filtering was applied. The trajectories of all markers were imported into Matlab® (The MathWorks Inc., Natic, MA, USA) where all further analyses were carried out. In the walking trial, 20 consecutive steps (17.92 s corresponding to 4300 frames) were selected for the proof-of-principle analysis. In the balance trial, for simplicity, the same number of frames was used.

### Proof-of-principle analysis

Four aspects were assessed as proof-of-principle analysis: (1) how the prediction accuracy of the reconstruction algorithm depended on the position of the marker on the volunteer; (2) how the prediction accuracy depended on the number of frames available as input; (3) what influence the weight factor **w** used for neighboring markers had on the prediction accuracy; and (4) how the reconstruction accuracy depended on the number of PC-vectors that were used in the reconstruction. In addition, the prediction accuracy of the proposed algorithm was compared with the accuracy obtained in linear or cubic interpolation, which are the standard gap filling procedures in commercial motion tracking software. In each test, the trajectory of one selected marker was removed for a specified period from the matrix containing the trajectory of all markers. This matrix was submitted as input into the proposed reconstruction algorithm. The accuracy of the trajectory predicted by the reconstruction algorithm was then quantified by determining the absolute difference between the predicted and the measured (and then deleted) marker coordinates.

The assessment of the prediction accuracy in dependence of the marker’s position on the body was conducted for 8 markers on all major body segments, specifically for *LHEE, LANK, LKNE, LASI, LSHO, LELB, LWRA, and LFHD.* Three situations were tested: the trajectory of one selected marker was removed for the last 2 steps (3870 frames of the data available; 430 frames reconstructed); the last 10 steps (2150 frames available; 2150 frames reconstructed); or the last 18 steps (430 frames available; 3870 frames reconstructed) – corresponding to the situation of a marker falling off during an experiment. The same procedure was conducted in the balance trial. Differences between measured and predicted marker positions were listed as actual distance (mm) and also as percentage of the marker’s total range of motion (% ROM).

The prediction accuracy in dependence of varying length of the input trajectory was investigated for three selected markers, LKNE, LSHO, and LWRA. In the walking trial, the length of the input was varied between 1 step and 19 steps, and the remainder to 20 steps was reconstructed. In the balance trial, the length of the input trajectory was varied between 1 s and 17 s and the remainder of the 17.9-second balance trial was reconstructed. The mean and maximum Euclidean distance between the measured and predicted trajectories were calculated for the period in which the marker was missing.

The influence of the weighting factor assigned to neighboring markers was assessed using the same three markers as in the previous analysis (LKNE, LSHO, and LWRA). For both the walking and the balancing trial, 50% of the trajectory was provided as input, the other 50% were reconstructed. Neighboring markers, as defined in Tables 1–4, were assigned a weight between 0 and 30. Second nearest neighbors were assigned half of the weight of the direct neighbors. The prediction accuracy was quantified using the mean and the maximum Eucledean distance.

**Table 1:** Mean of the difference (absolute values) between predicted and measured marker trajectories in the walking trial.

WALKING MEAN ABSOLUTE DIFFERNCE				reconstructed based on 2 steps (input: 430 of 4300 frames)		reconstructed based on 10 steps (input: 2150 of 4300 frames)		reconstructed based on 18 steps (input: 3870 of 4300 frames)	
reconstructed marker	nearest markers (weight factor: 10)	second nearest markers (weight factor: 5)		absolute [mm]	% of ROM	absolute [mm]	% of ROM	absolute [mm]	% of ROM
***LHEE***	*LTOE*		X	8	1%	2	0%	2	<0.5%
	*LANK*		Y	5	5%	1	1%	1	1%
			Z	5	2%	1	0%	1	<0.5%
***LANK***	*LTOE*	*LKNE*	X	3	<0.5%	1	<0.5%	1	<0.5%
	*LHEE*		Y	2	2%	1	1%	1	1%
	*LTIB*		Z	3	2%	1	1%	1	1%
***LKNE*** +	*LTIB*	*LANK*	X	3	1%	2	<0.5%	1	<0.5%
	*LTHI*	*LASI*	Y	5	6%	1	1%	1	1%
			Z	3	4%	1	1%	1	1%
***LASI***	*RASI*		X	2	2%	1	1%	1	1%
	*LPSI*		Y	3	4%	2	3%	2	3%
	*RPSI*		Z	3	5%	1	2%	1	2%
***LSHO***	*LUPA*	*LELB*	X	2	2%	1	1%	<0.5	<0.5%
	*C7*		Y	1	1%	<0.5	<0.5%	<0.5	<0.5%
	*CLAV*		Z	1	2%	<0.5	<0.5%	<0.5	<0.5%
***LELB***	*LUPA*	*LSHO*	X	3	1%	2	1%	1	<0.5%
	*LFRA*	*LWRA*	Y	2	2%	2	2%	1	1%
		*LWRB*	Z	1	2%	1	<0.5%	2	<0.5%
***LWRA***	*LWRB*	*LELB*	X	5	1%	2	2%	2	2%
	*LFRA*		Y	4	3%	2	1%	2	2%
			Z	4	3%	1	1%	1	1%
***LFHD***	*RFHD*		X	2	2%	<0.5	<0.5%	<0.5	<0.5%
	*LBHD*		Y	1	1%	<0.5	<0.5%	<0.5	<0.5%
	*LBHD*		Z	1	2%	<0.5	<0.5%	<0.5	<0.5%

+ see Figure 1.

**Table 2:** Maximum of the difference (absolute values) between predicted and measured marker trajectories in the walking trial.

WALKING MAXIMUM ABSOLUTE DIFFERNCE				reconstructed based on 2 steps (input: 430 of 4300 frames)		reconstructed based on 10 steps (input: 2150 of 4300 frames)		reconstructed based on 18 steps (input: 3870 of 4300 frames)	
reconstructed marker	nearest markers (weight factor: 10)	second nearest markers (weight factor: 5)		absolute [mm]	% of ROM	absolute [mm]	% of ROM	absolute [mm]	% of ROM
***LHEE***	*LTOE*		X	31	4%	11	1%	10	1%
	*LANK*		Y	14	13%	5	5%	4	4%
			Z	13	6%	5	2%	4	2%
***LANK***	*LTOE*	*LKNE*	X	8	1%	4	1%	4	1%
	*LHEE*		Y	7	7%	4	4%	2	2%
	*LTIB*		Z	8	5%	3	2%	2	1%
***LKNE*** +	*LTIB*	*LANK*	X	11	3%	7	2%	4	1%
	*LTHI*	*LASI*	Y	16	18%	5	6%	4	5%
			Z	10	13%	5	7%	3	4%
***LASI***	*RASI*		X	10	9%	6	5%	3	3%
	*LPSI*		Y	16	20%	11	14%	6	8%
	*RPSI*		Z	10	18%	5	9%	3	5%
***LSHO***	*LUPA*	*LELB*	X	7	7%	3	3%	2	2%
	*C7*		Y	2	2%	1	1%	1	1%
	*CLAV*		Z	6	10%	2	3%	1	2%
***LELB***	*LUPA*	*LSHO*	X	13	6%	7	3%	4	2%
	*LFRA*	*LWRA*	Y	7	6%	6	5%	5	4%
		*LWRB*	Z	5	9%	3	5%	2	3%
***LWRA***	*LWRB*	*LELB*	X	20	5%	18	4%	13	3%
	*LFRA*		Y	21	16%	8	6%	7	5%
			Z	13	9%	5	4%	4	3%
***LFHD***	*RFHD*		X	20 *	23% *	19 *	22% *	19 *	22% *
	*LBHD*		Y	7 *	9% *	9 *	11% *	9 *	11% *
	*LBHD*		Z	4	8%	2	4%	1	2%

+ see Figure 1.

* artifact in the original data (see Figure 2).

**Table 3:** Mean of the difference (absolute values) between predicted and measured marker trajectories in the one-leg balance trial.

ONE-LEG BALANCE MEAN ABSOLUTE DIFFERNCE				reconstructed based on 1.8 s balancing (input: 430 of 4300 frames)		reconstructed based on 9.0 s balancing (input: 2150 of 4300 frames)		reconstructed based on 16.1 s balancing (input: 3870 of 4300 frames)	
reconstructed marker	nearest markers (weight factor: 10)	second nearest markers (weight factor: 5)		absolute [mm]	% of ROM	absolute [mm]	% of ROM	absolute [mm]	% of ROM
***LHEE*** *	*LTOE*		X	7	37%	<0.5	<0.5%	1	5%
	*LANK*		Y	1	4%	<0.5	<0.5%	<0.5	<0.5%
			Z	4	10%	<0.5	<0.5%	<0.5	<0.5%
***LANK*** *	*LTOE*	*LKNE*	X	2	12%	<0.5	<0.5%	<0.5	<0.5%
	*LHEE*		Y	2	8%	<0.5	<0.5%	<0.5	<0.5%
	*LTIB*		Z	3	7%	<0.5	<0.5%	<0.5	<0.5%
***LKNE*** *	*LTIB*	*LANK*	X	3	19%	<0.5	<0.5%	<0.5	<0.5%
	*LTHI*	*LASI*	Y	1	5%	<0.5	<0.5%	<0.5	<0.5%
			Z	2	14%	<0.5	<0.5%	<0.5	<0.5%
***LASI***	*RASI*		X	2	9%	2	9%	1	5%
	*LPSI*		Y	1	5%	1	5%	<0.5	<0.5%
	*RPSI*		Z	1	9%	1	9%	1	9%
***LSHO***	*LUPA*	*LELB*	X	1	4%	<0.5	<0.5%	<0.5	<0.5%
	*C7*		Y	1	3%	<0.5	<0.5%	<0.5	<0.5%
	*CLAV*		Z	<0.5	<0.5%	<0.5	<0.5%	<0.5	<0.5%
***LELB***	*LUPA*	*LSHO*	X	2	7%	<0.5	<0.5%	<0.5	<0.5%
	*LFRA*	*LWRA*	Y	1	4%	<0.5	<0.5%	<0.5	<0.5%
		*LWRB*	Z	1	6%	<0.5	<0.5%	<0.5	<0.5%
***LWRA***	*LWRB*	*LELB*	X	5	23%	1	5%	1	5%
	*LFRA*		Y	2	9%	<0.5	<0.5%	<0.5	<0.5%
			Z	1	10%	1	10%	<0.5	<0.5%
***LFHD*** +	*RFHD*		X	1	3%	1	3%	<0.5	<0.5%
	*LBHD*		Y	4	13%	<0.5	<0.5%	<0.5	<0.5%
	*LBHD*		Z	1	8%	<0.5	<0.5%	<0.5	<0.5%

* One-leg stance: the volunteer was standing on the right leg, the left foot was in the air.

+ see Figure 3.

**Table 4:** Maximum of the difference (absolute values) between predicted and measured marker trajectories in the one-leg balance trial.

ONE-LEG BALANCE MAXIMUM ABSOLUTE DIFFERNCE				reconstructed based on 1.8 s balancing (input: 430 of 4300 frames)		reconstructed based on 9.0 s balancing (input: 2150 of 4300 frames)		reconstructed based on 16.1 s balancing (input: 3870 of 4300 frames)	
reconstructed marker	nearest markers (weight factor: 10)	second nearest markers (weight factor: 5)		absolute [mm]	% of ROM	absolute [mm]	% of ROM	absolute [mm]	% of ROM
***LHEE*** *	*LTOE*		X	18	94%	2	10%	2	10%
	*LANK*		Y	4	17%	1	4%	1	4%
			Z	8	19%	1	2%	1	2%
***LANK*** *	*LTOE*	*LKNE*	X	5	30%	1	6%	1	6%
	*LHEE*		Y	4	17%	2	8%	1	4%
	*LTIB*		Z	7	17%	2	5%	1	2%
***LKNE*** *	*LTIB*	*LANK*	X	7	45%	4	26%	2	13%
	*LTHI*	*LASI*	Y	4	21%	3	16%	1	5%
			Z	4	28%	2	14%	1	7%
***LASI***	*RASI*		X	6	28%	5	24%	2	9%
	*LPSI*		Y	4	21%	2	11%	1	5%
	*RPSI*		Z	2	18%	2	18%	2	18%
***LSHO***	*LUPA*	*LELB*	X	2	8%	2	8%	1	4%
	*C7*		Y	3	10%	1	3%	1	3%
	*CLAV*		Z	1	13%	1	13%	1	13%
***LELB***	*LUPA*	*LSHO*	X	6	22%	3	11%	1	4%
	*LFRA*	*LWRA*	Y	3	12%	1	4%	1	4%
		*LWRB*	Z	2	13%	1	6%	1	6%
***LWRA***	*LWRB*	*LELB*	X	10	45%	3	14%	3	14%
	*LFRA*		Y	4	19%	1	5%	1	5%
			Z	4	38%	2	19%	1	10%
***LFHD*** +	*RFHD*		X	4	14%	2	7%	1	3%
	*LBHD*		Y	9	30%	1	3%	1	3%
	*LBHD*		Z	3	25%	1	8%	1	8%

* One-leg stance: the volunteer was standing on the right leg, the left foot was in the air.

+ see Figure 3.

Reconstruction accuracy in dependence of the number of PC-vectors used in the reconstruction was also investigated using LKNE, LSHO, and LWRA in the walking and the balancing trial. Similar to the weighting factor analysis, 50% of the marker trajectory was provided as input, the other 50% were reconstructed. The number of PC-vectors used for the reconstruction varied between 5 and 111.

The proposed algorithm was compared to interpolation as gap filling technique in a series of additional tests. The walking trial was selected for this analysis and an artificial gap of varying length was created in the LKNE marker. Interpolation techniques require data before and after the gap. The gap was, therefore, not created at the end of the file, but started in the middle of the dataset (arbitrarily chosen). The length of the gap was stepwise increased by 4 frames starting from a gap of 2 frames up to a gap of 98 frames. Matlab’s functions for linear and cubic interpolation and the gap filling algorithm proposed in the current paper were used to fill these gaps. The accuracy of each of the three gap filling techniques was quantified by calculating the mean Euclidean distance between the reconstructed and measured marker positions.

## Results

**Figure 1:**
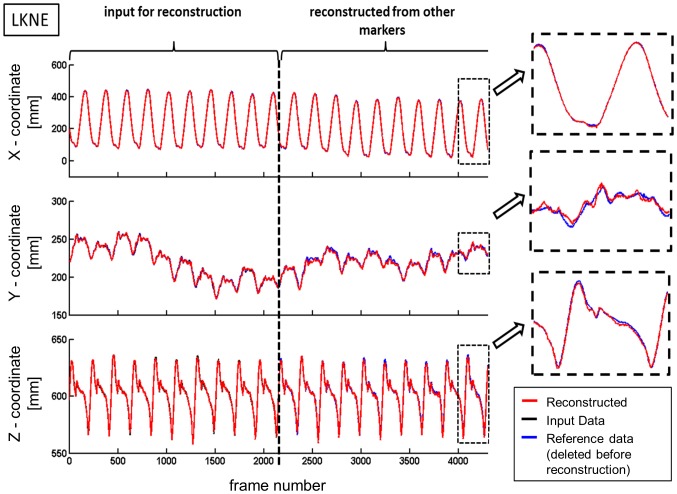
Trajectory of the left knee marker (LKNE) recorded for 20 steps of walking. In this example, the first 10 steps (frame 1-2150) of the measured LKNE-coordinates were used as input data for the reconstruction, the following 10 steps (frame 2151-4300) were reconstructed.

**Figure 2:**
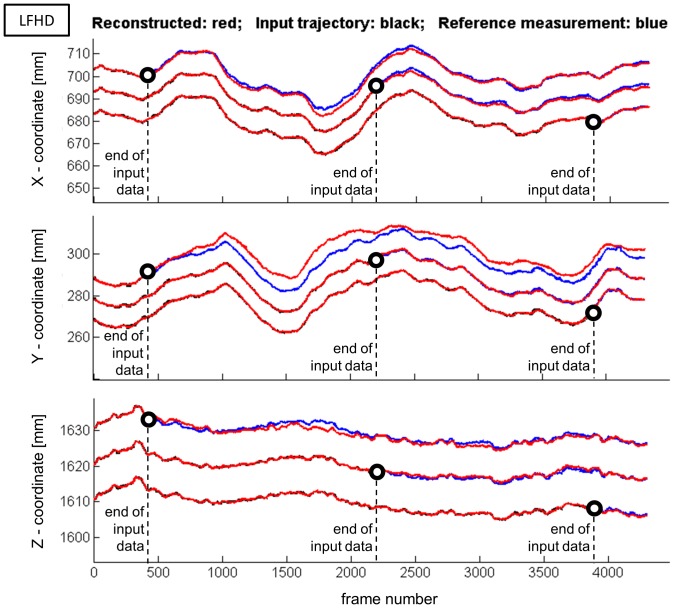
Trajectory of the left front head marker (LFHD) recorded during a one-leg balance trial. The underlying black line represents the input data for the reconstruction, the blue line represents the measured reference data that was reconstructed and the red line represents the reconstructed marker position. Three cases are shown: In the lower line, 3870 frames (90% of the data) were provided as input data for the reconstruction, 430 frames were reconstructed. The middle line (shifted by +10 mm in each coordinate) shows the case where 2150 frames (50% of the data) were provided as input, 2150 frames were reconstructed. The top line (shifted by +20 mm) shows the case where only 430 frames (10% of the data) were provided as input and 3870 frames were reconstructed. The broken lines and the black circles indicate the first frame from which on the trajectories were reconstructed.

In both, the walking trial (Figure 1) and the balance trial (Figure 2), good qualitative agreement was observed between predicted and measured marker trajectories. The predicted marker positions followed the general characteristics of the measured trajectory, even if only 10% of the trajectory was provided as input for the reconstruction (Figure 2, Tables 1–4).

**Figure 3:**
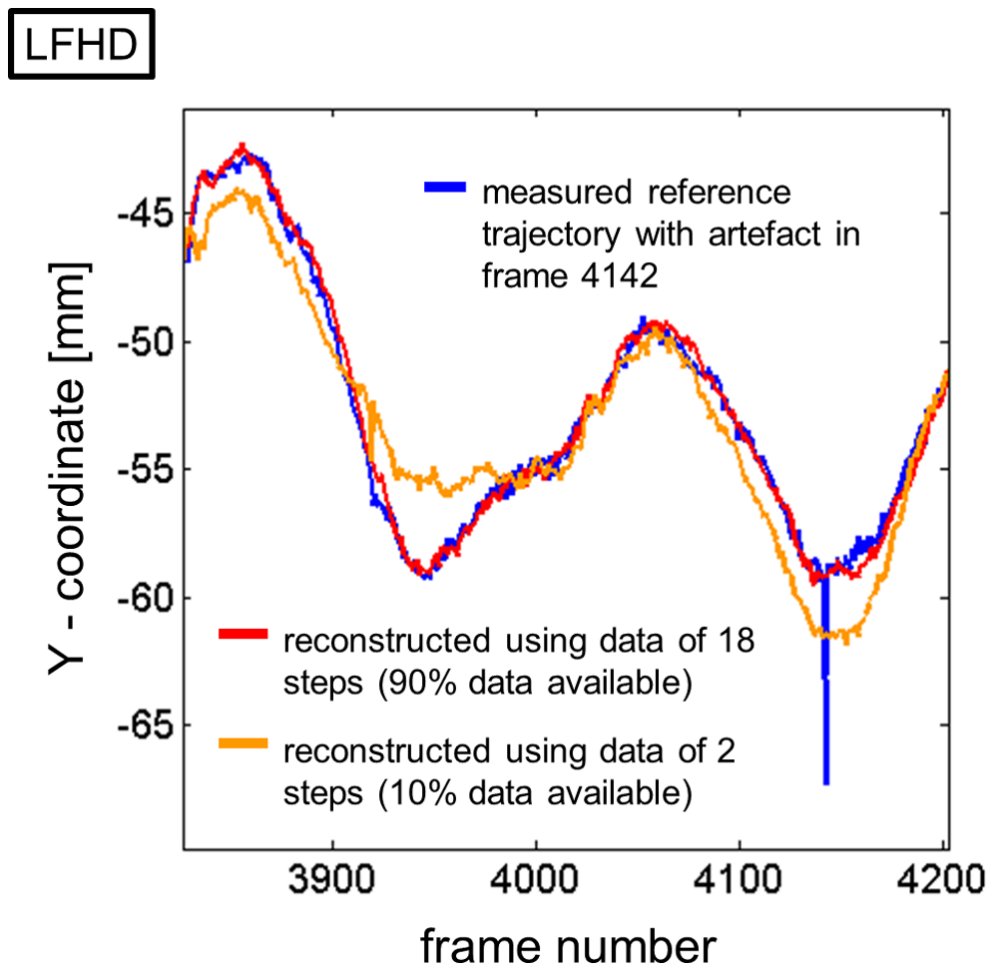
Detail of the Y-coordinate of the left front head marker (LFHD) with a measurement artifact. The artifact is visible in the measured trajectory (blue line) at frame 4142. The results of the reconstruction based on 2 steps or 18 steps are displayed as orange or red lines, respectively.

In the walking trial, the absolute difference between measured and reconstructed positions of the 8 selected markers, averaged over the all reconstructed values, varied between 1 and 8 mm, between 0.5 and 2 mm, and between 0.5 and 2 mm for reconstructions with input data available from 2, 10, or 18 steps, respectively (Table 1). This corresponded to between 0.5% and 6%, 0.5% and 3%, and 0.5% and 3% of the entire range of motion of the reconstructed markers. The largest differences were observed in markers placed on the most distal body segments (*LHEE*, *LWRA*). The maximal differences observed for the three reconstruction conditions were 31 mm, 19 mm, and 19 mm (Table 2), However, it is important to note that the differences may not only stem from inaccuracies in the reconstruction, but may also be caused by artifacts in measured trajectories (Figure 3).

In the balance trial, the averaged absolute differences between measured and reconstructed marker trajectories (Table 3) ranged from 0.5 to 7 mm, from 0.5 to 2 mm, and from 0.5 to 1 mm if 10%, 50%, or 90% of the marker trajectory were provided as input for the reconstruction. The maximum differences (Table 4) ranged from 1 to 18 mm, from 1 to 5 mm and from 1 to 3 mm, respectively.

**Figure 4:**
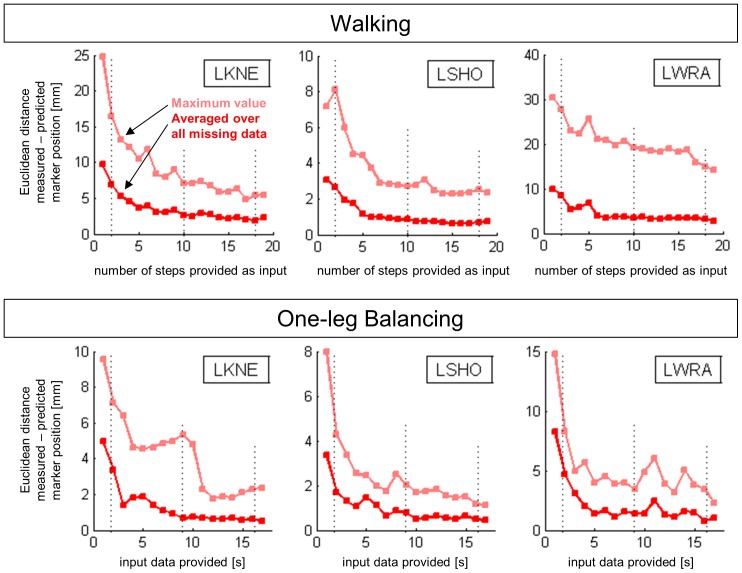
Reconstruction accuracy as a function of the amount of input data available for the reconstruction. LKNE: left knee, LSHO: left shoulder, and LWRA: left wrist marker; walking (top) and balancing (bottom). The broken vertical lines indicate the three situations used to create Tables 1–4.

The reconstruction accuracy was in all analyzed markers generally improved if more input data was provided (Figure 4), however, in all markers some fluctuations and deviations from this general trend were observed. This suggests that any additional data provided as input has the potential to alter the PCA solution, and consequently the reconstruction.

**Figure 5:**
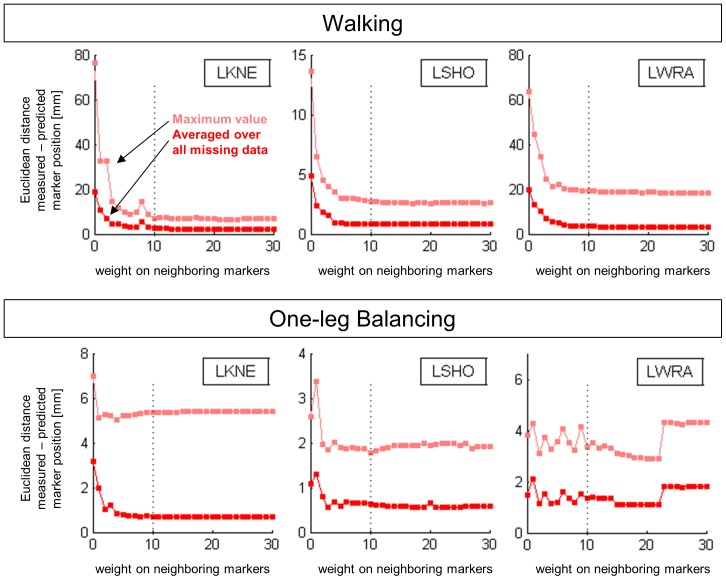
Reconstruction accuracy as a function of the weighting factor assigned to neighboring markers. LKNE: left knee, LSHO: left shoulder, and LWRA: left wrist marker. The broken vertical line indicates the weighting factor of 10, which was used as standard for all other analyses in the current study.

The analysis of the impact of the weighting factor on neighboring markers on the reconstruction accuracy (Figure 5) showed that no or small (<5) weighting factors generally produced substantially worse results than larger weighting factors. In the two datasets used in the current proof-of-principal analysis, a weighting factor of 10 appeared to be a good compromise between exploiting the higher correlation of the movement of neighboring markers and overemphasizing neighboring markers (and their noise levels and artifacts).

**Figure 6:**
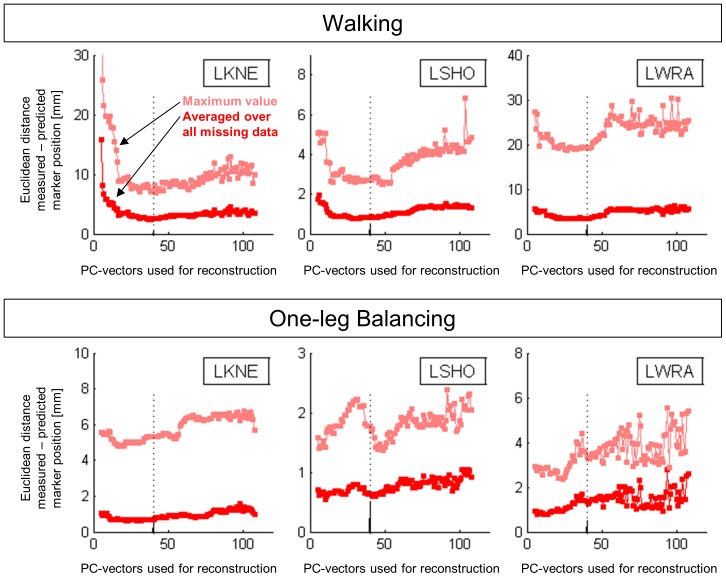
Accuracy if the reconstruction was based on a selected number of PC-vectors. (LKNE: left knee, LSHO: left shoulder, and LWRA: left wrist marker). The broken vertical line highlights the results obtained with 40 PC-vectors, which was used as standard for all other analyses in the current study.

The comparison of the reconstruction accuracy between reconstructions based on a different number of PC-vectors (Figure 6) showed roughly a U-shaped response. If too few PC-vectors were used, then the features of the movement were insufficiently represented by the PCA and the reconstruction result suffered. However, if more than 50 PC-vectors were used, then the difference between predicted and measured trajectory also started to increase gradually. The 40 PC-vectors selected for the analyses in the current study represented more than 99.98% of the variance in the walking trial and more than 99.94% of the variance in the balance trial. The shapes of the graphs shown in Figure 6 suggest that 40 PC-vectors are well suited for walking trials. In the balance trials, where the signal-to-noise ratio is much lower due to the low motion amplitude, a smaller number of PC-vectors may in some cases lead to a better reconstruction.

**Figure 7:**
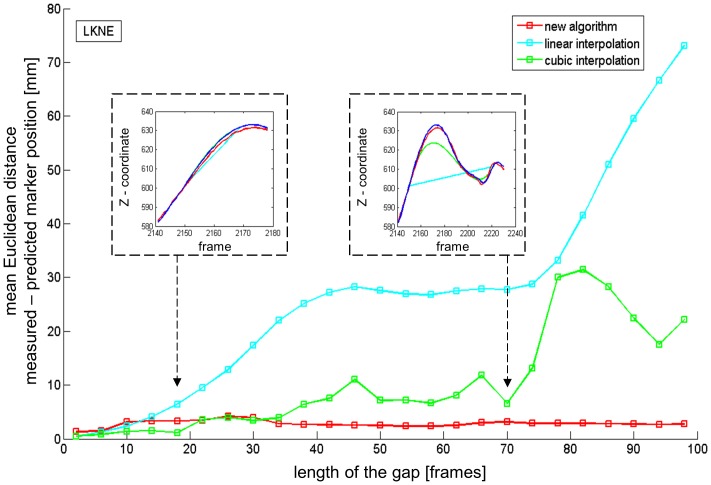
Comparison of the proposed reconstruction algorithm with interpolation based gap filling techniques. The main graph shows the mean Eucledian distance between measured and reconstructed marker positions for various gap lengths. The two inserted graphs show as an example the z-coordinate of the trajectories at the position of the gap for the two indicated gap lengths (18 and 70 frames).

In the comparison of the proposed gap filling algorithm with interpolation techniques (Figure 7), small gaps of 10 frames or less were more accurately filled with linear or cubic interpolation techniques. For gap lengths up to 30 frames, cubic interpolation provided an equal or better result than the proposed algorithm. However, the prediction accuracy of the linear or cubic interpolation technique quickly deteriorated if the gap length exceeded 10 frames or 30 frames, respectively. The average distance between the measured trajectory and the one predicted by the proposed algorithm remained lower than 4 mm for all tested gap lengths between 2 and 98 frames.

## Discussion

In both of the two essential types of human motion tested in the current study, gait and postural sway, good agreement was found between predicted and actually measured marker coordinates. The results of the current analysis, therefore support the underlying assumption that strong intercorrelations exist between the movements of different markers in human motion and that PCA can be used to quantitatively characterize these internal constraints [24].

Even if only 2 steps of walking or 1.8 s of postural sway (10% of the whole marker data) were provided as input in the current study, the reconstructed marker trajectory differed on average no more than 11 mm from the originally measured trajectory. The reconstruction result improved further to average distances below 5 mm if the marker trajectory was available more than 50% of the trial. The difference between reconstructed and measured trajectory was thus smaller than the cross section of the markers (20 mm); smaller than soft tissue artifacts (16 mm [30]; 21 to 31 mm [31]); small compared to the jumps or ripples that can sometimes be observed when the number of cameras detecting a marker changes or when reflections interfere (typically 5–10 mm [e.g. Figure 3]); and comparable to the alterations in marker trajectories produced by low-pass filtering [32], [33], which typically follows as a next post-processing step after gap-filling.

The systematic analysis of how the reconstruction result improves when a weight factor is used on neighboring markers showed that this is an effective and important tool to improve the reconstruction accuracy. In some cases, the average difference between measured and predicted marker positions improved by one order of magnitude. The availability of neighboring markers is higher for proximally positioned markers and offers one explanation for the observation that the best reconstruction results were obtained for proximal markers (e.g. LSHO). The more distal the markers were positioned (LHEE, LWRA) the worse was the reconstruction result, but the more it benefitted from increasing the weight factor on the few available neighboring markers.

The systematic analysis of how the reconstruction accuracy depended on the number of PC-vectors used in the reconstruction demonstrated that the ability of the PCA to separate correlated signals in the data from uncorrelated noise can be utilized to further improve the reconstruction result. The results of this study suggest that the optimal number of PC-vectors to include may depend on the type of movement – probably due to a different signal-to-noise ratio in the marker data.

Compared to other gap-filling approaches, the proposed algorithm differs in some important prerequisites, advantages, and disadvantages. A fundamental conceptual prerequisite and limitation is that the PCA has to be able to adequately characterize the interrelation between the marker movements. Hence, it is important to note that, firstly, sufficient input data needs to be available. A mathematical prerequisite is that more frames need to be available than dimensions in the posture vector. Moreover, the results of the current study suggest that several cycles in a cyclic motion or a few sway cycles in balance trials should be represented in the input data. Secondly, no changes that could alter the interrelation between the segment motions may occur in the subject’s movement patterns. The algorithm is well suited for continuous or cyclic motion patterns, however, it is a priori not suited for datasets that quantify events (e.g. external perturbation experiments) and its suitability for trials with transition phases (e.g. walking-running, change of direction maneuvers) remains to be tested; Thirdly, the performance of the suggested algorithm depends on the employed marker set. The more information about the motion of the subject is available, the better the performance of the proposed gap-filling algorithm. Marker sets representing only a fraction of the motion (e.g. only on the lower extremities) will reduce the accuracy of the reconstruction.

If these prerequisites are met, then there are also a number of advantages compared to other gap-filling routines. For example, in contrast to interpolation techniques, the gaps may be positioned anywhere within the trajectory including at the beginning or end of the file and they may contain several movement cycles (i.e. several extreme values). In contrast to Kalman filter approaches [3]–[6], the method does not suffer from drift. Moreover, the proposed method is little affected by noise, outliers or artifacts in the available sections of the reconstructed trajectory. However, outliers or artifacts particularly in the adjacent markers may affect the reconstruction accuracy. The method is conceptually related to data-driven pattern recognition methods [8]–[11] and achieved similar reconstruction accuracy [8], however, a separate database with similar movement data is not required and the optimization or data-mining techniques (e.g. nearest neighbor analysis) employed by these methods are replaced by a simple coordinate transformation. It is possible that replacing the PCAs with non-linear directional statistical tools might further improve the reconstruction, however, further research is necessary to investigate this speculation.

## Concluding Remarks

The result of the proof-of-principle analysis presented in the current study confirmed the general applicability of the proposed gap-filling algorithm. Estimating missing information is always tenuous, however, in many situations in human motion analysis it cannot be entirely avoided. For some of these situations, particularly for trials with multiple repetitions of similar movement patterns such as in balance or fatigue trials, the proposed reconstruction method may provide a better solution than the methods that have been previously applied. Furthermore, it is likely that the results of the reconstruction could be further improved, if more information, for example, ground reaction forces were included.

Situations in which gaps occur in more than one marker have not been considered in the current proof-of-principle analysis. It seems likely that the approach presented here may also be useful if multiple markers are missing, however, some additional challenges will need to be solved, such as, if gaps occur in neighboring markers. The optimal solution for multiple missing markers is, therefore, likely to be dependent on the specific situation and may include a combination of the proposed algorithm with other gap-filling procedures.

The underlying concept that was applied here to solve the so called “missing marker problem” may also be applicable when addressing related challenges in motion analysis. For example, to determine what unique information each marker contributes in an analysis, to detect and remove artifacts, to study coupling of movement over joints, or to detect a change in the movement pattern.

## Supporting Information

Dataset S1 This.zip-file contains two Matlab scripts (.m-files) and two datasets (.mat files). The file “PredictMissingMarkers.m” is an implementation of the algorithm described in the current paper. The file “Script_to_test_PredictMissingMarkers.m” is a script that allows an assessment of the proposed algorithm by (i) reading in test data, (ii) creating gaps in the test data, (iii) filling the gaps by calling the function “PredictMissingMarkers”, and (iv) comparing the reconstructed marker trajectories with the original trajectories. Two datasets are provided as test data: “OneLegBalancing_18seconds_noGaps.mat” and “Walking_20steps_noGaps.mat”.
(ZIP)

Text S1 This pdf file contains a copy of the Matlab script for “PredictMissingMarkers”. It is intended to give readers who use other software than Matlab an idea how the algorithm may be implemented.
(PDF)
