# Supplementary material for: A Novel Approach to Solve the “Missing Marker Problem” in Marker-Based Motion Analysis That Exploits the Segment Coordination Patterns in Multi-Limb Motion Data
Source: PLoS One. 2013 Oct 30;8(10):e78689. doi: 10.1371/journal.pone.0078689 (PMC3813748; doi:10.1371/journal.pone.0078689)
Supplement: Text S1 — This pdf file contains a copy of the Matlab script for “PredictMissingMarkers”. It is intended to give readers who use other software than Matlab an idea how the algorithm may be implemented. (PDF) [file pone.0078689.s002.pdf]

```
function [ ReconstructedFullDataSet ] = ...
    PredictMissingMarkers( Data_gaps, nearNeighbours,...
                                      secondaryNeighbours,...
                                      weights_nearNeighbours,...
                                      weights_secondaryNeighbours)
% PredictMissingMarkers
% searches for gaps ("NaN") in the marker coordinates and fills these gaps
% using the natural correlation structure in the marker coordinates.
%
% Input:
% Data_gaps: matrix with marker data organized in the form
%            [ x1(t1), y1(t1), z1, x2, y2, z2, x3, ..., zn(t1)
%              x1(t2), y1(t2), ...
%               ...                                   ...
%              x1(tm), y1(tm), ...                   , zn(tm)]
% nearNeighbors, secondaryNeighbours: if it is known which markers have
%            gaps, then neighbouring markers can be pointed out to
%            improve the prediction (see paper)
% weights_nearNeighbours, weights_secondaryNeighbours: the weights on
%            neighbouring markers can be modified manually (see paper).
%            The recommended default values are 10 and 5 for near and
%            secondary neighbours, respectively.
%
%
% Output:
% ReconstructedFullDataSet: Matrix in the same form as the input matrix
%                           with columns that had gaps replaced by a
%                           reconstructed marker trajectory
%
% Limitations: There are types of motion analysis data where this code
%              should not be used and there are a number of additional
%              limitations.
%              Please find more information in the paper.
%
%

% (c) Peter Federolf, 2013, version 2.0, all rights reserved.
% If you use this code for research - in the current or in a modified
% version - then please cite the corresponding paper:
% Federolf P.: A novel approach to solve the “missing marker problem”
% in marker-based motion analysis that exploits the segment coordination
% patterns in multi-limb motion data. Plos One. (submitted 2013)

%% Variable Definitions

columns = size(Data_gaps,2);
frames  = size(Data_gaps,1);

define_weights = ones(1,columns/3);

if nargin==1
elseif nargin==2
    for i = 1:size(nearNeighbours,2)
        define_weights(nearNeighbours(i))= 10;
    end
    % if weight inputs are empty, then set to default
```

```matlab
elseif nargin==3
    for i = 1:size(nearNeighbours,2)
        define_weights(nearNeighbours(i))= 10;
    end
    for i = 1:size(secondaryNeighbours,2)
        define_weights(secondaryNeighbours(i))= 5;
    end
    % if weight inputs are empty, then set to default

elseif nargin==4
    for i = 1:size(nearNeighbours,2)
        define_weights(nearNeighbours(i))= weights_nearNeighbours;
    end
    for i = 1:size(secondaryNeighbours,2)
        define_weights(secondaryNeighbours(i))= 5;
    end
elseif nargin==5
    for i = 1:size(nearNeighbours,2)
        define_weights(nearNeighbours(i))= weights_nearNeighbours;
    end
    for i = 1:size(secondaryNeighbours,2)
        define_weights(secondaryNeighbours(i))= weights_secondaryNeighbours;
    end
end



%% Step 1: Detect which columns have gaps and where
columns_with_gaps = find(any(isnan(Data_gaps),1));
frames_with_gaps = find(any(isnan(Data_gaps),2));


%% Step 2: center the data by subtracting a mean trajectory,
%          then define the matices needed for the prediction

Data_without_compromized_markers = Data_gaps;
Data_without_compromized_markers(:,columns_with_gaps)=[];

mean_trajectory.x = ...
    mean(Data_without_compromized_markers(:,...
                            1:3:columns-size(columns_with_gaps,2) ),2);
mean_trajectory.y = ...
    mean(Data_without_compromized_markers(:,...
                            2:3:columns-size(columns_with_gaps,2) ),2);
mean_trajectory.z = ...
    mean(Data_without_compromized_markers(:,...
                            3:3:columns-size(columns_with_gaps,2) ),2);

Data_centered(:,1:3:columns) = ...
    Data_gaps(:,1:3:columns)-...
        repmat(mean_trajectory.x,1,columns/3);
Data_centered(:,2:3:columns) = ...
    Data_gaps(:,2:3:columns)-...
        repmat(mean_trajectory.y,1,columns/3);
```

```
Data_centered(:,3:3:columns) = ...
    Data_gaps(:,3:3:columns)-...
        repmat(mean_trajectory.z,1,columns/3);


M__ = Data_centered;

M__zeros = M__;
M__zeros(:,columns_with_gaps)=0;

N__no_gaps = Data_centered( ~any(isnan(M__),2),:);

N__zeros = N__no_gaps;
N__zeros(:,columns_with_gaps) = 0;


%% Step 3: normalization:
%          all markers are treated as if they carry the same
%          amount of information: normalization to unit variance.
%          Then markers are multiplied with a weight vector that may
%          emphasise adjacent markers for higher precision.


mean_N__no_gaps    = mean(N__no_gaps,1);
mean_N__zeros = mean(N__zeros,1);
stdev_N__no_gaps   = std(N__no_gaps,1,1);


M__zeros = (M__zeros - repmat(mean_N__zeros,size(M__zeros,1),1))./...
                       repmat(stdev_N__no_gaps,size(M__zeros,1),1).*...
                       repmat(reshape([1 1 1]'*define_weights,1,[]),...
                               size(M__zeros ,1),1);

N__no_gaps =(N__no_gaps-repmat(mean_N__no_gaps,size(N__no_gaps ,1),1))./...
                        repmat(stdev_N__no_gaps,size(N__no_gaps ,1),1).*...
                        repmat(reshape([1 1 1]'*define_weights,1,[]),...
                                       size(N__no_gaps ,1),1);

N__zeros = (N__zeros - repmat(mean_N__zeros,size(N__zeros ,1),1))./...
                       repmat(stdev_N__no_gaps,size(N__no_gaps ,1),1).*...
                       repmat(reshape([1 1 1]'*define_weights,1,[]),...
                                       size(N__no_gaps ,1),1);


%% Step 4: Perform a PCA on the incomplete and full Markersets

function PCA_result = PCA( Data )

n_eig = min(40,size(Data,2)-3);
opts.v0 = ones(size(Data,2),1);

% compute covariance matrix on time series
% c = cov(Data,1);
```

```matlab
c = Data'*Data / size(Data,1);

%Eigenvalue decomposition
[v,EV] = eigs(c,n_eig,'lm',opts );

% build the output structure
PCA_result.Eigenvectors = v;
%PCA_result.Eigenvalues = diag(EV);
end

PCA__no_gaps = PCA(N__no_gaps);
PC_vectors__no_gaps = PCA__no_gaps.Eigenvectors;

PCA__zeros = PCA(N__zeros);
PC_vectors__zeros = PCA__zeros.Eigenvectors;


%% Step 5: Calculate Transformation Matrix for Principal Movements
%          Transform Data first into incomplete-, then into full-PC basis
%          system.

T__ = PC_vectors__no_gaps' * PC_vectors__zeros;


ReconstructedData = M__zeros*PC_vectors__zeros*T__*PC_vectors__no_gaps';
    % Equation 1 in the paper


%% Step 6: Reverse normalization

ReconstructedData = repmat(mean_N__no_gaps,frames,1)+ReconstructedData.*...
    repmat(stdev_N__no_gaps,size(ReconstructedData ,1),1)./...
    repmat(reshape([1 1 1]'*define_weights,1,[]),...
                           size(M__zeros ,1),1);




%% Step 7: Add mean trajectory subtracted in step 2 to obtain original
%          dataset + missing marker

ReconstructedData(:,1:3:columns) = ...
    ReconstructedData(:,1:3:columns) + ...
    repmat(mean_trajectory.x,1,columns/3);
ReconstructedData(:,2:3:columns) = ...
    ReconstructedData(:,2:3:columns) + ...
    repmat(mean_trajectory.y,1,columns/3);
ReconstructedData(:,3:3:columns) = ...
    ReconstructedData(:,3:3:columns) + ...
    repmat(mean_trajectory.z,1,columns/3);
```

```
%% Prepare Output
%
ReconstructedFullDataSet = Data_gaps;
for j = columns_with_gaps
    ReconstructedFullDataSet(:,j) = ReconstructedData(:,j);

end


end  % function
```
